# Cytokinins beyond plants: synthesis by *Mycobacterium tuberculosis*

**DOI:** 10.15698/mic2015.05.203

**Published:** 2015-05-04

**Authors:** Marie I. Samanovic, K. H. Darwin

**Affiliations:** 1 New York University School of Medicine, Department of Microbiology, New York, NY 10016 USA.

**Keywords:** Mycobacterium tuberculosis, proteasome, nitric oxide, LONELY GUY, cytokinins

## Abstract

*Mycobacterium tuberculosis* (*M. tuberculosis*) resides mainly inside macrophages, which produce nitric oxide (NO) to combat microbial infections. Earlier studies revealed that proteasome-associated genes are required for *M. tuberculosis* to resist NO via a previously uncharacterized mechanism. Twelve years later, we elucidated the link between proteasome function and NO resistance in *M. tuberculosis *in *Molecular Cell*, 57 (2015), pp. 984-994. In a proteasome degradation-defective mutant, Rv1205, a homologue of the plant enzyme LONELY GUY (LOG) that is involved in the synthesis of phytohormones called cytokinins, accumulates and as a consequence results in the overproduction of cytokinins. Cytokinins break down into aldehydes that kill mycobacteria in the presence of NO. Importantly, this new discovery reveals for the first time that a mammalian bacterial pathogen produces cytokinins and leaves us with the question: why is *M. tuberculosis*, an exclusively human pathogen, producing cytokinins?

 Tuberculosis remains a major global health challenge and is one of the world’s deadliest communicable diseases, causing an estimated 9 million new cases and 1.5 million deaths in 2013. Tuberculosis is caused by *M. tuberculosis*, a pathogen residing mainly within macrophages. However, *M. tuberculosis *persists despite the production of NO and other molecules that repress microbial growth. In 2003, in an effort to find new targets for treatment of the disease, the laboratory of Carl Nathan published the results of a screen for NO-sensitive *M. tuberculosis *mutants and discovered that mycobacterial proteasome-associated components are essential for NO resistance. Since this discovery, many of the components required for proteasomal degradation were identified in *M. tuberculosis*, and every enzyme involved in the mycobacterial proteasome system is essential for the NO resistance and virulence of this pathogen. However, one question remained unanswered: how does proteolysis protect the bacteria against NO toxicity?

Our recent work finally solved this mystery thanks to a genetic approach. We hypothesized that the accumulation of one or more proteasome substrates was responsible for NO sensitivity in the proteolysis mutants. Surprisingly we discovered that the accumulation of a single protein, Rv1205, a newly identified proteasome substrate, accounts for the NO sensitivity of an *M. tuberculosis* proteolysis mutant. Thus, disruption of the gene Rv1205 in a proteolysis mutant restored NO resistance to wild type levels, and also restored a significant amount of bacterial growth in mice.

Most interestingly, we established that Rv1205 is a homologue of LONELY GUY (LOG), a plant enzyme that converts *N^6^*-modified adenosine monophosphate derivatives into *N^6^*-modified adenine free bases called cytokinins. Using metabolomics we observed the accumulation of at least one cytokinin breakdown product in a proteolysis-deficient *M. tuberculosis* strain and determined that this and other aldehyde products of cytokinin breakdown synergize with NO to kill *M. tuberculosis*.

Unexpectedly, our work revealed for the first time that a non-phytopathogen produces cytokinins. Indeed,* M. tuberculosis* is an exclusively human pathogen, which is never found in the environment. This leaves us wondering what role cytokinins play during tuberculosis infections.

In plants, cytokinins are master regulators of growth and development. They act as signaling molecules at very low concentrations both locally and distal from the site of production. They are perceived in plants via two-component signaling phosphorelays that are similar to those used by bacteria to sense and respond to environmental stimuli. We speculate that cytokinins could possibly be involved in a similar regulatory system controlling gene expression in *M. tuberculosis. *Perhaps cytokinins are used as communication molecules amongst mycobacteria to control the development of an infection. In plants, the first component of the cytokinin-signaling cascade is the binding of cytokinins to cyclases/histidine kinases-associated sensing extracellular (CHASE) domain proteins, which mediate signal transduction. To date, there is no such receptor known in *M. tuberculosis,* and CHASE domains are absent from its proteome. Therefore, whether or not *M. tuberculosis* can sense cytokinins is an open question.

**Figure 1:**
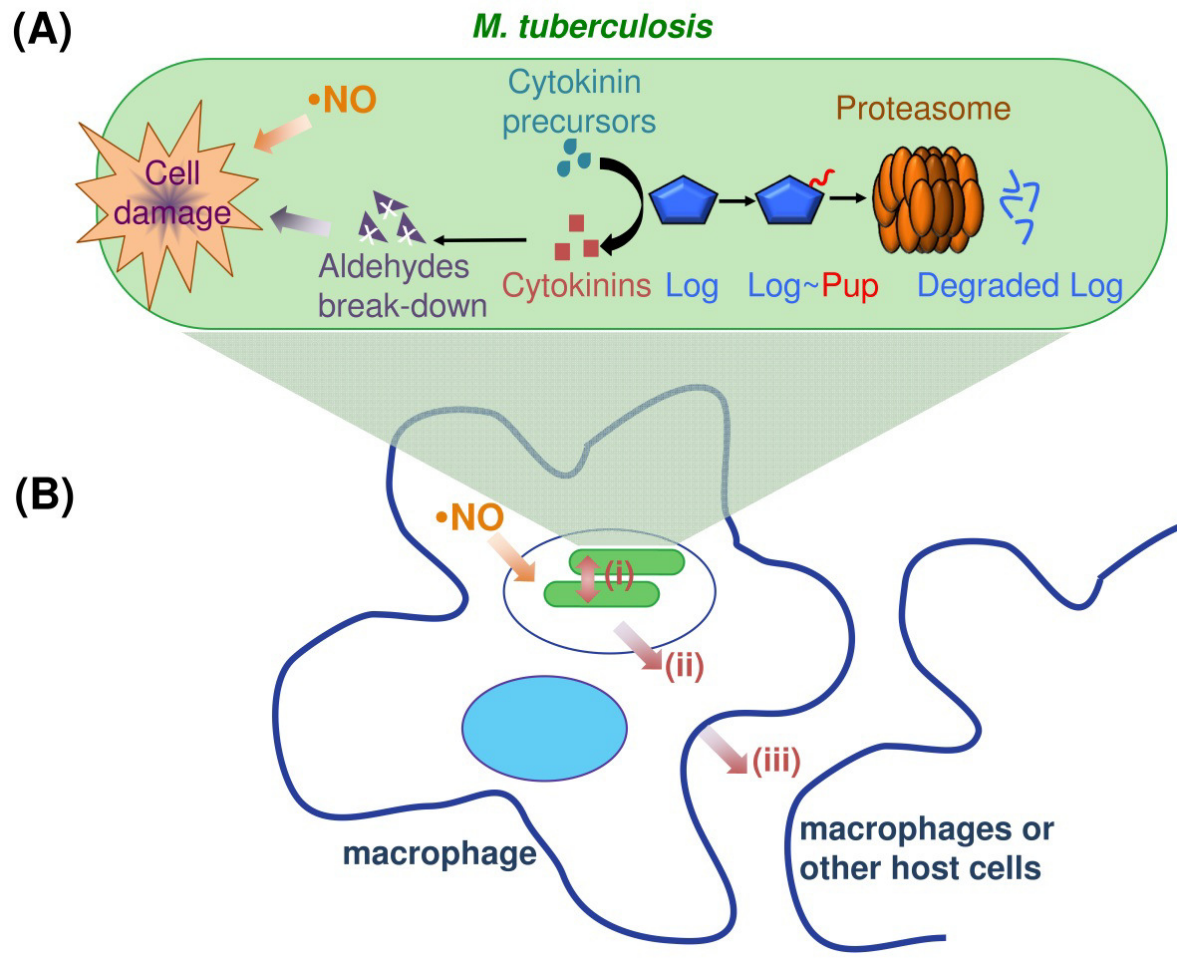
FIGURE 1: Cytokinin synthesis in *M. tuberculosis*. **(A)** *M. tuberculosis* Log is tightly regulated by the mycobacterial proteasome to maintain low amounts of the protein. In a proteolysis-deficient mutant, Log accumulates and results in elevated cytokinin levels, the breakdown of which into aldehydes kills mycobacteria in the presence of NO produced by macrophages. **(B) ** To date, the role of cytokinins in *M. tuberculosis* infection is unknown. Cytokinins may be used as signaling molecules to communicate among mycobacteria (i) and/or act on the host (ii, iii) in order to establish a successful infection.

Equally, we speculate that cytokinins may be secreted by *M. tuberculosis *to act on the host in order to facilitate infection. In plant systems, phytobacteria produce cytokinins as a tool for invasion. For example, *Rhodococcus fascians*, a distant relative of *M. tuberculosis*, secretes cytokinins to stimulate proliferation of plant tissue in order to establish its infection niche. Currently, we do not know if cytokinins have an effect on human cells.

*M. tuberculosis* “Log" levels are tightly controlled post-translationally by the proteasome, suggesting that the bacteria probably finely tune cytokinin production. This limiting step may allow a specific distribution of the hormones at the right place and at the right time. Accordingly, *M. marinum*, a mycobacterium species that infects aquatic animals, carries a *log* homologue, the expression of which is detected in the granulomas of infected frogs but not in cultured macrophages.

The cytokinin biosynthesis pathway in *M. tuberculosis* remains to be elucidated*. *In plants, cytokinins are mainly synthesized *de novo*, and tRNA degradation is also a minor source of cytokinins. We have yet to determine the provenance of cytokinin precursors in *M. tuberculosis.*

Finally, with the discovery of *M. tuberculosis* *log, *we found that other bacterial species harbor LOG homologues. Many of these species, such as *Staphylococcus aureus*, are important human pathogens. This reinforces the exciting notion that cytokinins have roles beyond plant development that have yet to be characterized.

